# Taxonomic Identification and Nutritional Analysis of *Pterocladiella capillacea* in Zhanjiang

**DOI:** 10.3390/md23010011

**Published:** 2024-12-28

**Authors:** Zhengwen Lv, Hongyan Cai, Nenghui Li, Hang Li, Jun Zeng, Kefeng Wu, Luming Deng, Huaqiang Tan, Hua Ye

**Affiliations:** 1 The Affiliated Dongguan Songshan Lake Central Hospital, Guangdong Medical University, Dongguan 523326, China; lzw922@outlook.com (Z.L.); lnh19966591137@163.com (N.L.); 2 Southern Marine Science and Engineering Guangdong Laboratory (Zhanjiang), Oyster Industrial Technology Institute of Zhanjiang, Zhanjiang 524059, China; caihongyan@zjblab.com (H.C.); lih@zjblab.com (H.L.); jzeng15@163.com (J.Z.); 3 Marine Biomedical Research Institution, Guangdong Medical University, Zhanjiang 524023, China; winokhere@sina.com (K.W.);

**Keywords:** red algae, *P. capillacea*, molecular identification, nutritional composition

## Abstract

To evaluate the nutritional value and development potential of *Pterocladiella capillacea* in the marine environment of Naozhou Island, Zhanjiang, this study conducted species classification and identification, followed by an analysis of key nutritional components. The combination of morphological and molecular results confirmed the identification of the collected samples as *P. capillacea*. Further analysis showed that *P. capillacea* in Zhanjiang had a moisture content of 74.9% and a protein content of 24%. In comparison, the fat (0.4%) and carbohydrate (15.4%) contents were relatively low, with moderate ash (14.3%) and crude fiber (9.1%) content. It contains a diverse range of fatty acids, with saturated fatty acids accounting for 51.82% and unsaturated fatty acids accounting for 48.18% of the total. The amino acid composition was also diverse, with essential amino acids comprising 31.58% and flavor-enhancing amino acids constituting 54.85%. The minerals contained four major elements and four trace elements, while heavy metal levels were within safety limits, ensuring their edibility. In conclusion, *P. capillacea* is a high-protein, low-fat economic seaweed with a favorable amino acid and fatty acid composition, rich in minerals, and with significant nutritional and developmental potential. This study provides important data to support future research and utilization of this seaweed.

## 1. Introduction

*Pterocladiella capillacea* belongs to the phylum Rhodophyta, class Florideophyceae, order Gelidiales, family Pterocladiaceae, and genus *Pterocladiella* [1,2]. This species is common in countries such as China, France, Italy, Greece, Spain, Israel, and Portugal. Currently, there are 26 species of *Pterocladiella* algae worldwide that are widely distributed in warm temperate to tropical regions [3]. In China, five species of the genus *Pterocladiella* have been identified, predominantly distributed along the coastal areas of Shandong, Zhejiang, Fujian, Guangdong, Guangxi, and Hainan provinces, primarily found on the rocky substrates in the low-tide zone and on the stones in the mid-tide zone [4,5]. As a global species, it serves as an important raw material for producing high-quality agar, which is characterized by high gel strength and low gel temperature, making it commonly used in biomedical and biotechnological applications [6,7,8,9]. Currently, it is commercially developed as a major seaweed resource in regions such as Portugal and Israel [10]. Additionally, it has widespread applications in the nutritional supplement and pharmaceutical industries and is a significant source of polyunsaturated fatty acids, proteins, dietary fibers, amino acids, balanced mineral ratios, vitamins, pigments, and volatile compounds [11,12]. Research has also shown that its extracts exhibit biological activities, such as anti-inflammatory, antioxidant, cytotoxic, and antitumor effects, indicating that it is a marine macroalgae with development potential [13,14,15].

Zhanjiang, located at the southern tip of mainland China and bordered by the South China Sea to the east, boasts abundant macroalgae resources, with cyclically fluctuating biomass. These rich seaweed resources provide ample raw materials for biological research and resource utilization, serving as a foundation for further studies. Due to the shared evolutionary history, ecological adaptability, and similar habitat requirements, the morphological structure of *P. capillacea* in Zhanjiang is particularly similar to that of *Ge lidium* sp., making it difficult to distinguish between them based solely on morphological characteristics [2,16]. With the widespread application of molecular techniques for the classification and identification of seaweeds, the limitations of traditional morphological classification methods have been effectively addressed [4,17,18]. Accurate species identification ensures the reliability of analytical results and prevents confusion arising from nutritional value differences between species. It also lays a solid foundation for subsequent nutritional component assessment and food safety research. Therefore, this study employed a combined morphological and molecular approach to identify the species of *P. capillacea* in Zhanjiang and analyze its primary nutritional components, providing valuable data for future scientific research and resource utilization of *P. capillacea* in this region.

## 2. Results

### 2.1. Morphological Features of P. capillacea

**Figure 1:**
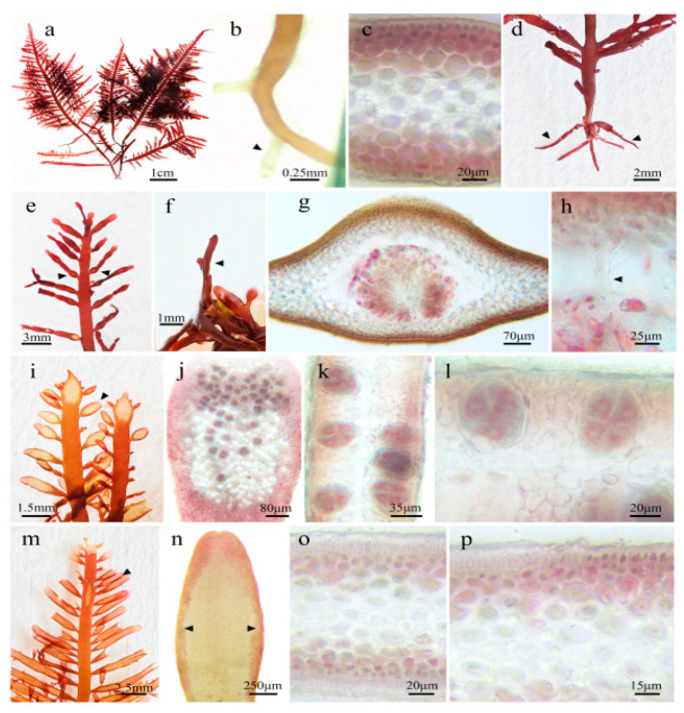
Morphology and anatomical structure of *P. capillacea*. Collected from the Naozhou Island, Zhanjiang. Note: (**a**) Morphological appearance of *P. capillacea*; (**b**) multiple rhizoids growing from the base of *P. capillacea*; (**c**) longitudinal section of the *P. capillacea*; (**d**) erect axis emerging from the prostrate branch; (**e**) reproductive thallus bearing receptacles; (**f**) surface view of a receptacle; (**g**) cross-section of a mature receptacle; (**h**) conceptacles within a mature receptacle showing paraphyses and nutritive filaments; (**i**) thallus bearing tetrasporangia; (**j**) surface view of tetrasporangia; (**k**,**l**) cross-sectional views of tetrasporangia; (**m**) apex of a branch bearing antheridia; (**n**) small leaf bearing antheridia; (**o**,**p**) cross-sectional views of anther.

Morphology and Anatomy of *P. capillacea* (Figure 1): *P. capillacea* exhibits a purplish-red coloration with a cartilaginous texture, growing erectly, either singly or in clusters, reaching heights of 7–13 cm. The base of the thallus is anchored by tangled filiform holdfasts, from which one or more prominent feathered branches emerge along their apical main axis. The apices are spatulate or gradually tapering, contributing to an overall pyramidal outline characterized by regular bifurcations occurring 2–3 times. Branches typically arise opposite or alternate, tapering at the base and ending bluntly, with right-angled junctions between the main axis and branches; upper branches are denser, while lower branches are sparser (Figure 1a). *P. capillacea* develops numerous pseudoroots at its base (Figure 1b). Transverse sections through the mid-axis revealed a dense distribution of outer cortical cells, inner medullary cells, and root hair cells within the medulla (Figure 1c). The erect axis originates from the prostrate filaments (Figure 1d). Figure 1e illustrates the apical region of fertile branches, displaying paired arrangement of sporangial sori. Figure 1f exhibits the surface of sporangia, showing unilateral protuberances. Figure 1g presents a cross-sectional view of mature sporangia. Figure 1h details sporogenesis and nutritive filaments within mature sporangia. Figure 1i depicts the apical region of tetra sporangial branches of *P. capillacea*. Figure 1j displays the surface morphology of tetraspores. Figure 1k,l show cross-sections of tetrasporangia, revealing distinct cruciate divisions. Figure 1m shows the apical region of spermatangial branches, while Figure 1n illustrates spermatangial sori. Figure 1o,p display cross-sections of spermatangia, featuring elongated cortical cells on the outer surface. These observations provide a detailed description of the morphological characteristics of *P. capillacea*, laying the groundwork for further analysis and understanding.

### 2.2. Analysis of Cox1 Gene Sequence in P. capillacea

**Figure 2:**
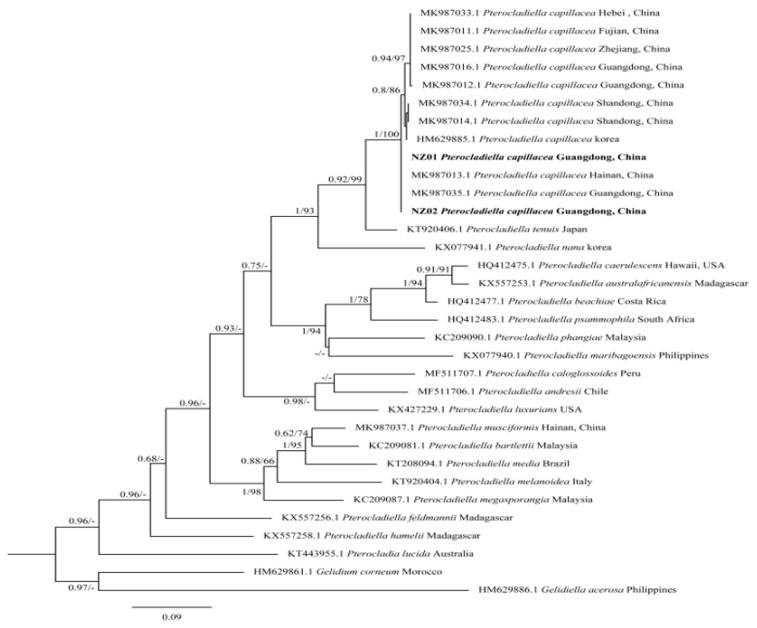
Maximum likelihood tree of *P. capillacea* based on the cox1 gene. Note: The numbers on the branches represent Bayesian posterior probabilities and maximum likelihood bootstrap values, from left to right. The bold text indicates newly amplified sequences from this study. Only nodes with Bayesian posterior probabilities ≥ 0.60 and maximum likelihood bootstrap values ≥ 60 are shown in the figure.

Using *Gelidium corneum* and *Gelidiella acerosa* as outgroups, we constructed a phylogenetic tree of the *cox1* gene with 33 sequences: 31 from GenBank and 2 newly generated sequences. The final length of the sequence was 546 bp. Phylogenetic trees were constructed using both ML and BI, yielding similar topologies. Therefore, we present a phylogenetic tree constructed using the ML method (Figure 2), with support values at each node indicating BI and ML values, respectively, from left to right or top to bottom.

The results showed that the two samples of *P. capillacea* from Naozhou Island, Zhanjiang formed a single clade. These two sequences, NZ01 and NZ02, were grouped with sequences from Hainan Province (MK987013.1) and Guangdong Province (MK987035.1) in the same branch. There were no nucleotide differences between the *cox1* gene sequences of the two samples, and the Bayesian posterior probability and maximum likelihood bootstrap values were both 1 and 100%, respectively. The combined analysis of the morphological and molecular identification results confirmed that both NZ01 and NZ02 were *P. capillacea*.

### 2.3. Analysis of Basic Nutritional Components in P. capillacea

The general nutritional composition of *P. capillacea* was analyzed, and its major nutritional components were compared with those of several common economically important seaweeds. Table 1 presents the results. Compared to macroalgae such as *Cymogongrus flabelliformis*, *Chondria crassicaulis* (Rhodophyta), *Dictyota dichotoma*, *Sargassum naozhouense* (Phaeophyceae), and *Monostroma nitidum* (Chlorophyta), *Pterocladiella capillacea* (Rhodophyta) exhibited higher moisture (74.9%) and protein (24%) contents, but lower fat (0.4%) and carbohydrate (15.4%) contents. The ash (14.3%) and crude fiber (9.1%) contents were moderate.

### 2.4. Amino Acid Composition, Content, and Nutritional Analysis of P. capillacea

**Figure 3:**
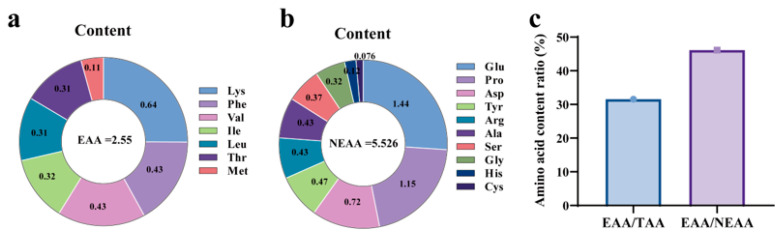
Amino acid composition and content of *P. capillacea.* Note: (**a**) represents essential amino acids, (**b**) represents non-essential amino acids, and (**c**) represents the ratio of amino acid contents.

Figure 3 presents the amino acid analysis results for *P. capillacea*, indicating a total amino acid content (TAA) of 8.076%, which includes seven essential amino acids (EAAs) accounting for 2.55% and ten non-essential amino acids (NEAAs) comprising 5.526%. NEAA constituted 68.42% of the TAA, while EAA represented 31.58%, approaching the FAO/WHO standard of approximately 40% for EAA/TAA. The ratio of EAA to NEAA was 46.15%. Among the non-essential amino acids, glutamic acid (Glu), proline (Pro), and aspartic acid (Asp) were present in high concentrations, with glutamic acid and proline accounting for 32.07% of the total amino acids, making them significant components of the NEAA profile. Taste-active amino acids, which include savory and sweet amino acids, accounted for 54.85% of the TAA, with flavor-active amino acids (FAAs) included Asp and Glu, and sweet amino acids (SAAs) included Gly, Ala, Ser, and Pro. The assessment of the protein amino acids in *P. capillacea* is presented in Table 2. Both AAS (amino acid score) and CS (Chemical Score) analyses indicated that Leucine (Leu) is the first limiting amino acid in *P. capillacea*. The AAS ranged from 18.45 to 62.5, and the CS ranged from 15.02 to 40.32.

### 2.5. Analysis of Fatty Acid Composition of P. capillacea

**Figure 4:**
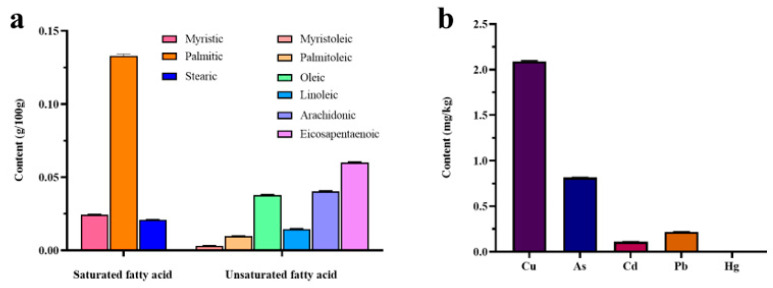
Fatty acid and heavy metal concentrations in *P. capillacea*. Note: (**a**) represents fatty acid content and (**b**) represents heavy metal content (Hg ≤ 0.01).

As shown in Figure 4a, *P. capillacea* is primarily composed of nine types of fatty acids, including three saturated fatty acids and six unsaturated fatty acids. The saturated fatty acid content was slightly higher (51.82%), with palmitic acid (38.65%) being the predominant saturated fatty acid followed by myristic acid (7.06%). The unsaturated fatty acid content was slightly lower (48.18%), mainly consisting of eicosapentaenoic acid (17.41%), arachidonic acid (11.71%), and oleic acid (10.99%).

### 2.6. Analysis of Major Element Content in P. capillacea

The contents of the main mineral elements in *P. capillacea* are presented in Table 3 and Figure 4b. The results indicated that the *P. capillacea* in Zhanjiang contained four major and four trace elements. Among the major elements, the calcium (Ca) content (7.97 mg/g) was similar to that of *Sargassum naozhouense* and showed an increase of 212.5% compared to that of *Ulva lactuca*. K (21.2 mg/g) and Mg (2.40 mg/g) levels were lower than those in other seaweeds. In terms of trace elements, *P. capillacea* showed a low accumulation capacity for Zn (0.105 mg/g) and Fe (0.351 mg/g) but exhibited a particularly strong accumulation capacity for I (0.725 mg/g), which was significantly higher than that of *Ulva lactuca*. As shown in Figure 4b, the heavy metal contents in *P. capillacea* are as follows: copper (Cu) 2.09 mg/kg, arsenic (As) 0.82 mg/kg, cadmium (Cd) 0.11 mg/kg, lead (Pb) 0.22 mg/kg, and mercury (Hg) 0.01 mg/kg. According to the GB 19643-2016 “National Food Safety Standards for Algae and Its Products” regarding pollutant limits (Pb ≤ 1.0 mg/kg, Hg ≤ 0.5 mg/kg, As ≤ 1.5 mg/kg) and the EU 2023/915 regulations on maximum limits for food contaminants (Cd ≤ 3.0 mg/kg), the heavy metal levels of *P. capillacea* in Zhanjiang complied with the established standards.

## 3. Discussion

*P. capillacea,* as a cosmopolitan species, is commonly utilized in the fields of biomedical science, biotechnology, and the nutritional supplements and pharmaceutical industries, serving as a significant source of various nutrients [3,11]. Accurate taxonomic identification is fundamental for further research on algae. Combining morphological observations with molecular biological methods enhances the precision of classification and identification results [24]. The morphological data from this study indicate that *P. capillacea* has a purplish-red appearance and cartilaginous texture, growing upright either individually or in clusters, with a height of 7–13 cm. The base of the alga is secured by entangled filamentous holdfasts consisting of one or more distinct pinnate branches and a central axis. The transverse section of the central axis revealed a medulla composed of more than a dozen layers of loosely arranged cells, with flattened cells and thick cell walls in the medulla. Tetrasporangia sacs are primarily dispersed within the cortical layer of the alga, with mature tetrasporangia sacs that are round or oval in shape and divided in a cruciform manner. This was consistent with the descriptions provided by Wang et al. [4] and R. F. Patarra et al. [3]. Subsequently, a phylogenetic tree was constructed using ML methods to verify the identification. By combining morphological and molecular identification techniques, the collected samples were confirmed to be *P. capillacea*.

Today, seaweeds have gradually become a natural resource rich in nutritional components, including high-quality proteins, dietary fibers, polysaccharides, polyunsaturated fatty acids, minerals, vitamins, pigments, and various phytochemicals, such as polyphenols [25,26,27]. However, these nutrients are often influenced by various factors such as species, geographical origin, season, environmental conditions, and physiological changes [28,29,30]. Research has shown that the nutritional composition of different types of seaweed varies significantly. The protein content of green and red algae (ranging from 10.0% to 47.0%) was generally higher than that of brown algae (3.0% to 17.0%), except brown algae *Undaria pinnatifida*, whose protein levels ranged from 11% to 24%, although most brown algae species have protein levels below 15% [26,31]. This study analyzed six basic nutritional components of *P. capillacea* collected from the coast of Zhanjiang: moisture, protein, fat, ash, crude fiber, and carbohydrates. As shown in Table 1, the protein content of *P. capillacea* (24%) is slightly higher than that of *D. dichotoma* (10.38%), *S. naozhouense* (13.95%), and *M. nitidum* (9.29%), and is comparable to the protein content of other red algae such as *Gelidium microdon* (23.4%) [11]. However, Paiva et al. [11] indicated that the basic nutritional composition of *P. capillacea* in the Azores region is as follows: moisture 86%, protein 20.16%, fat 4.32%, carbohydrates 19.76%, and ash 10.69%. Among these, the fat content was higher than that of *P. capillacea* in Zhanjiang (0.4%), whereas the other components were similar. The differences between the above results may be attributed to factors such as the analytical methods, geographical location, and harvest time.

Amino acids are fundamental building blocks of proteins, playing a crucial role in muscle and tissue repair, regulating metabolism, immune function, and the synthesis of enzymes and hormones, as well as maintaining normal physiological functions [32]. Some amino acids must be obtained from dietary sources because the human body cannot synthesize them. These functions make amino acids vital for sustaining energy levels, promoting healing, and supporting overall health [33,34]. *P. capillacea* along the coast of Zhanjiang contains seventeen amino acids, including seven essential amino acids (EAAs) required by the human body and six flavor amino acids. Among EAAs, lysine (Lys) is the most abundant, while glutamic acid (Glu) is the most abundant non-essential amino acid (NEAA), followed by proline (Pro). The results of this study showed that the EAA/TAA ratio was 31.58% and the EAA/NEAA ratio was 46.15% in *P. capillacea*. According to the ideal protein pattern recommended by the FAO/WHO, the amino acid composition of high-quality proteins should have an EAA/TAA ratio of around 40% and an EAA/NEAA ratio of over 0.6 [35]. Thus, the amino acid composition of proteins in *P. capillacea* is slightly below the requirements of this ideal pattern, indicating a slight deficiency in the quality of the amino acid composition. The amino acid score of a protein evaluates whether the content of each essential amino acid meets the body’s needs; the closer the score is to 100, the more it indicates that the protein meets human nutritional requirements [36]. However, the amino acid scores of the seaweeds were generally low. For instance, Dawczynski et al. [37] calculated the amino acid score of *Laminaria* sp. to be 31.4, whereas *Sargassum fusiforme* (formerly *Hizikia fusiformis*) (Phaeophyceae) had an amino acid score of 40. The amino acid score of *P. capillacea* in Zhanjiang also reflected a significant difference when compared to the standard amino acid pattern. Therefore, it is essential to complement its amino acid profile by pairing it with that of other food sources to achieve balanced amino acid nutrition.

Most seaweeds have low lipid content, ranging from 0.3% to 7.0%, and generally exhibit a high content of unsaturated fatty acids. They are particularly rich in n-3 and n-6 series polyunsaturated fatty acids (PUFAs), making them an important source of PUFAs [38,39]. Levy et al. [40], in a study exploring the distribution of fatty acids in large red algae, found that in Gelidiales species, the primary polyunsaturated fatty acids were 20:4ω6 and 20:5ω3. In contrast, only 20:4ω6 was found in the Gracilariales species. *P. capillacea* in Zhanjiang is primarily composed of nine fatty acids, including three saturated fatty acids and six unsaturated fatty acids. Palmitic acid was the predominant saturated fatty acid, whereas unsaturated fatty acids mainly consisted of eicosapentaenoic acid (17.41%) and arachidonic acid (11.71%). Research indicates that palmitic acid not only helps maintain the physical properties of cell membranes but also regulates the body’s physiological functions. However, excessive intake of palmitic acid is not advisable because it may increase the risk of cardiovascular diseases and lead to abnormalities in blood lipids, hyperglycemia, and fat accumulation [41,42]. Among all ω-3 fatty acids, eicosapentaenoic acid (EPA) has shown potential efficacy for certain psychiatric disorders and helps improve the symptoms of depression [43,44,45]. Arachidonic acid (AA), as a precursor for the synthesis of inflammatory mediators such as prostaglandins and leukotrienes, has been found to play a significant role in metabolic processes and is being explored as a potential therapeutic target for cardiovascular disease treatment [46].

Mineral elements play a crucial role in maintaining human health and normal physiological functions [47]. In this study, *P. capillacea* in Zhanjiang demonstrated a rich mineral content, exhibiting accumulation capabilities for potassium (K) (21.20 mg/g), calcium (Ca) (7.97 mg/g), and iodine (I) (0.725 mg/g), while showing relatively lower accumulation for other elements, making it a valuable source of K, Ca, and I. Research indicates that K regulates intracellular osmotic pressure and the acid–base balance of body fluids and participates in the metabolism of sugars and proteins [48]. Ca is essential for bone formation, contributes to blood cell synthesis, and regulates muscle contraction, nerve transmission, and blood clotting [49]. Iodine is a critical component of thyroid hormones, such as thyroxine (T3 and T4), which regulate metabolism, promote growth and development, and maintain body temperature, thus playing an essential role in the normal development of the brain and nervous system in infants [50]. Heavy metals, including copper, mercury, cadmium, lead, and arsenic, are toxic elements that can adversely affect the nervous, organ, immune, and reproductive systems, potentially increasing the cancer risk [51,52]. There are numerous reasons for heavy metal contamination in seaweeds, including marine pollution, bioaccumulation by marine organisms, climatic factors, and geological factors [53,54]. Regular monitoring of heavy metal content is essential to ensure the safe consumption of seaweed, along with measures to reduce environmental heavy metal pollution. In this study, the heavy metal content of *P. capillacea* in Zhanjiang was within safe limits, confirming its edibility and safety for consumption.

## 4. Materials and Methods

### 4.1. Algal Strain and Culture Conditions

**Figure 5:**
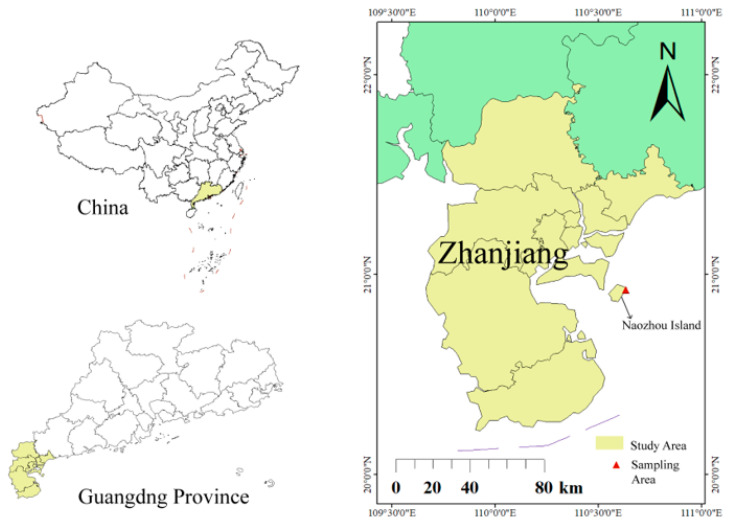
Collection sites of fresh samples from China.

*P. capillacea* samples used in this study were collected from Donghaitou, Naozhou Island, Zhanjiang, Guangdong Province, China (110°62′09″ E, 20°88′79″ N) in April 2024 (Figure 5). Healthy and intact thalli in their peak growth phases were selected and transported to the laboratory in oxygenated containers. The samples were rinsed with sterilized seawater to remove surface contaminants and temporarily maintained in an algal culture room under the following conditions: a temperature of 25 °C, salinity of 30‰, light intensity of 80 μmol/m^2^/s, and a photoperiod of 12 h:12 h (L:D), with aeration.

### 4.2. Morphological Characteristics and Cox1 Gene Sequence Analysis

Healthy intact *P. capillacea* thalli were randomly selected from temporary holding tanks for morphological observation. Fresh samples were spread on white trays, and thallus lengths were measured using a ruler. The overall appearance, color, texture, branching patterns, and cystocarp shapes were examined using a stereomicroscope (Daoyi Di S65,Guangzhou, China). Hand sections of characteristic parts were prepared, stained with acetic acid carmine solution, and observed under a microscope to examine the size and number of cortical and medullary cells and their arrangement. The layers of cortical cells, absorptive filaments, spermatangia, and tetrasporangia were observed using an upright fluorescence microscope (LEICA DM6 B, Leica Microsystems, Wetzlar, Germany). The observations were recorded and photographed.

Total DNA was extracted from *P. capillacea* using the Plant Genome Rapid Extraction Kit (Tiangen Biotech, Beijing, China) following the manufacturer’s instructions. The extracted DNA served as a template for the amplification of the *cox1* gene sequence using the GWSFn/Cox IR1 primer pair. The PCR conditions were set as follows: initial denaturation at 94 °C for 3 min, followed by 35 cycles of 94 °C for 30 s, 49.5 °C for 40 s, and 72 °C for 50 s, with a final extension at 72 °C for 5 min. PCR products were assessed via 1% agarose gel electrophoresis and subsequently cloned and sequenced by BGI Genomics (Guangzhou, China). The obtained sequences were subjected to BLAST searches in the NCBI database, and the relevant sequences were downloaded (GenBank accessionnumbers: PQ682581, PQ682582). A total of 33 cox1 gene sequences were used to construct the phylogenetic tree. The tree was built using maximum likelihood (ML) and Bayesian Inference (BI) methods. The ML analysis was performed using MEGA v.7.0.21 software, with the best evolutionary model selected by Modeltest and involved 1000 bootstrap replicates. The BI analysis was conducted using MrBayes v.3.1.2, utilizing the Markov Chain Monte Carlo (MCMC) method, running for 1,000,000 generations with sampling every 100 generations, and discarding the first 25% of samples as burn-in. Posterior probabilities for each branch were calculated.

### 4.3. Nutrient Composition Analysis

A suitable amount of seaweed collected from Donghaitou, Naozhou Island, was selected from the seaweed temporary holding room for nutritional analysis of *Pterocladiella capillacea*. The analysis included the determination of moisture, ash, protein, crude fat, amino acid type and content, fatty acid composition and content, and mineral element content. Three parallel tests were performed for each nutritional component. Moisture content was measured according to GB 5009.3-2016 (Method 1), ash content by GB 5009.4-2016 (Method 1), protein content by GB 5009.5-2016, crude fat by GB 5009.6-2016 (Method 1), and amino acid content following GB/T 5009.124-2016, “National Food Safety Standard—Determination of Amino Acids in Food”.

### 4.4. Amino Acids Score Analysis

The amino acid content of *P. capillacea* was determined by the National Standard for Food Safety (GB5009.124-2016). The specific methodology involved the hydrolysis of the samples using acid, followed by centrifugation to obtain the amino acid solution. This solution was then subjected to HPLC (Nexis GC-2030, Shimadzu, Kyoto, Japan) analysis after appropriate derivatization. The content of each amino acid in the sample was calculated by comparison with the standard amino acid solution. The essential amino acids in *P. capillacea* were evaluated using AAS and CS based on the FAO/WHO-recommended ideal protein intake amino acid standards and the whole egg protein amino acid pattern [35]. The calculation formulae are as follows:

(1) a=(Amino acid content in the sample/Protein content in the sample)×100

(2) $$AAS=\left(\;\frac{a}{A}\;\right)\times 100$$

(3) $$CS\;=\left(\;\frac{a}{S}\;\right)\times 100$$

Note: *a*: the content of essential amino acids in the protein of the sample to be tested (mg/g); *A*: the corresponding content of essential amino acids according to the FAO/WHO standard scoring pattern (mg/g); *S*: the corresponding amino acid content in the amino acid scoring pattern of whole egg protein(mg/g).

### 4.5. Determination of Fatty Acid Composition and Content

The fatty acid content of *P. capillacea* was determined according to the National Food Safety Standard GB 5009.168—2016. The specific method is gas chromatography (GC). The procedure entails converting the fatty acids present in the samples to fatty acid methyl esters (FAMEs) through transesterification, followed by their separation and quantitative analysis using a gas chromatograph (Nexis GC-2030, Shimadzu, Kyoto, Japan). The type and quantity of fatty acids present in the samples were determined by comparison with established standards.

### 4.6. Determination of Main Element Content

The contents of elements such as Fe, Cd, Cu, Zn, As, Na, Ca, Mg, and K in *P. capillacea* were determined according to the National Food Safety Standard GB 5009.268-2016. The method employed is flame atomic absorption spectrometry (FAAS), which entails the wet digestion of the sample and using an appropriate acid, such as nitric acid, for this purpose. Subsequently, the digested solution is subjected to elemental analysis using flame absorption spectrometry (FAAS), thereby enabling the quantitative determination of the content of each element.

### 4.7. Statistical Analysis

In this study, the data obtained were analyzed and processed using the SPSS 22.0 software, and the results were presented statistically using mean ± standard deviation (Mean ± S.D.). Three biological replicates were established for the aforementioned experiments, and one-way ANOVA and Dunnett’s test verified the differences between groups. *p* < 0.05 indicates a significant difference, and *p* < 0.01 indicates a significant extreme difference. The mapping was conducted using GraphPad Prism v.8.0.2, MEGA v.7.0.21, MrBayes v.3.1.2, and ArcMap v.10.8.

## 5. Conclusions

Comprehensive data indicate that *P. capillacea* is a moisture-rich, high-protein, and low-fat economic seaweed. It has a favorable amino acid and fatty acid composition, is rich in minerals, and possesses a high nutritional value with certain health benefits. These characteristics suggest that *P. capillacea* has significant potential for development and utilization in both medicinal and dietary applications.

## Figures and Tables

**Table 1:** Nutritional composition of *P.capillacea* and other seaweeds %.

**Macroalgae Species**	**Moisture**	**Protein**	**Lipids**	**Ash**	**Crude fiber**	**Carbohydrates**
*Pterocladiella capillacea*	74.9	24	0.4	14.3	9.1	15.4
*Cymogongrus flabelliformis* [19]	14.24	11.75	0.21	21.4	5.82	52.4
*Chondria crassicaulis* [20]	25.56	11.06	0.26	7.16	12.29	55.96
*Dictyota dichotoma* [20]	12.54	10.38	2.85	10.36	5.45	63,87
*Sargassum naozhouense* [21]	-	13.95	2.4	41.79	10.88	29.37
*Monostroma nitidum* [22]	9.98	9.29	1.82	35.09	0.94	42.75

Notes: “-” indicates no data in the literature.

**Table 2:** Evaluation of essential amino acids composition in *P. capillacea*.

**EAA**	**FAO**	**Egg**	**Content**	**AAS**	**CS**
Lys	55	70	26.67	48.48	38.10
Leu	70	86	12.92	18.45 *	15.02 *
Phe + Tyr	60	93	37.50	62.50	40.32
Thr	40	47	12.92	32.29 **	27.48
Val	50	66	17.92	35.83	27.15 **
Ile	40	54	13.33	33.33	24.69

Note: * indicates the first limiting amino acid; ** indicates the second limiting amino acid.

**Table 3:** Mineral element content in *P. capillacea* mg/g.

**Macroalgae Species**	**Element**						
	**Ca**	**K**	**Mg**	**Na**	**Zn**	**Fe**	**I**
*P. capillacea*	7.97	21.20	2.40	2.24	0.105	0.351	0.725
*Cymogongrus flabelliformis* [19]	-	-	-	-	0.028	0.0003	-
*Chondria crassicaulis* [20]	-	-	-	-	0.019	0.511	-
*Sargassum naozhouense* [21]	11.23	126.75	-	-	0.083	2.739	-
*Ulva lactuca* [23]	2.55	-	16.45	-	0.13	2.72	0.04

Notes: “-” indicates no data in the literature.

## Data Availability

The original data presented in the study are included in the article; further inquiries can be directed to the corresponding author.
